# Phosphophoryn and Dentin Sialoprotein Effects on Dental Pulp Cell Migration, Proliferation, and Differentiation

**DOI:** 10.3390/dj6040070

**Published:** 2018-12-10

**Authors:** Shu-Feng Chuang, Yu-Hsuan Chen, Peter Ma, Helena H. Ritchie

**Affiliations:** 1 Institute of Oral Medicine, National Cheng Kung University, Tainan 709, Taiwan; sfchuang@mail.ncku.edu.tw (S.-F.C.); bella33.chen@gmail.com (Y.-H.C.); 2 Department of Stomatology, National Cheng Kung University Hospital, Tainan 709, Taiwan; 3 Department of Biomaterial Science, School of Dentitry, University of Michigan, Ann Arbor, MI 48109, USA; mapx@umich.edu; 4 Department of Cariology, Restorative Sciences and Endodontics, School of Dentistry, University of Michigan, Ann Arbor MI 48109, USA

**Keywords:** phosphophoryn (PP), dentin sialoprotein (DSP), dentinogenesis, dental pulp cells, cell migration, differentiation

## Abstract

Phosphophoryn (PP) and dentin sialoprotein (DSP) are two of the most abundant dentin matrix non-collagenous proteins, and are derived from dentin sialoprotein-phosphophoryn (DSP-PP) mRNA. Mutations in the DSP-PP gene are linked to dentinogenesis imperfecta II and III. Previously, we reported transient DSP-PP expression in preameloblast cells first, followed by co-expression in preameloblasts and young odontoblasts, and finally sustained expression in odontoblasts. This phenomenon raised the possibility that DSP/PP proteins secreted by preameloblasts might promote dental pulp cell migration toward the dental pulp border and promote dental pulp cell differentiation. To examine the effects of DSP/PP proteins on dental pulp cell development, we investigated:(1) native PP effects on dental pulpcell migration and matrix protein expression; and (2) recombinant DSP/PP protein effects on cell proliferation and differentiation. We found that PP promoted cell migration and the expression of high levels of Col type I and PP in dental pulp cells. The addition of recombinant DSP/PP proteins affected cell proliferation and differentiation in a dental pulp cell line. These findings strongly suggest that DSP/PP may modulate cell migration, cell proliferation and differentiation, thus leading to dentin formation. DSP/PP protein may be useful clinically for pulp tissue regeneration.

## 1. Introduction

Dentin sialoprotein (DSP) and phosphophoryn (PP) are two major dentin non-collagenous extracellular matrix proteins, which are derived from the dentinsialoprotein-phosphophoryn (DSP-PP; also termed dentinsialophosphoprotein, DSPP) gene. Briefly, DSP-PP mRNA is transcribed from the DSP-PP gene and then translated into DSP-PP precursor protein, which further undergoes cleavage by peptidasesbone morphogenetic protein (BMP1) and/or tolloid-related-1 (TLR1) to generate mature DSP and PP proteins [1,2,3]. DSP/PP represents the combination of DSP and PP proteins. PP plays an important role in the transition of predentin to dentin, which is necessary for the formation of mineralized teeth. To initiate this process, PP generates insoluble mineral aggregates by binding large amounts of calcium ions [4,5,6,7] and subsequently interacting with the “e” band of collagen to form a stable support for apatite crystallization [8]. Finally, the association of PP with collagen creates a nucleation site where mineralization can occur [8].

The transient expression of DSP protein and DSP-PP transcripts occurs first in the preameloblasts, and next in both preameloblasts and young odontoblasts. Finally, DSP-PP transcripts exhibitsustained expression in odontoblasts [9,10]. Since DSP-PP mRNA expression in preameloblasts could result in mature DSP and PP protein expression, we present DSP-PP protein expression as DSP/PP. It is unclear why DSP/PP first expresses in preameloblasts. However, it is unlikely that DSP/PP protein expression in preameloblasts participates in matrix mineralization. This possibility leads us to hypothesize that these two proteins might be able to induce dental pulp cell migration, odontoblast differentiation and subsequent dentin formation.

To better understand the potential role of transient DSP-PP mRNA or DSP/PP protein expression in preameloblasts, a rat dental pulp cell line (MRPC-1) was used to examine whether native PP could affect dental cell migration and matrix protein synthesis. We further investigated whether recombinant DSP/PP proteins could affect dental pulp M2H4 cell proliferation and cell morphology as well as DSP-PP mRNA expression.

## 2. Materials and Methods

### 2.1. PP Effect on Rat Dental Pulp Cell MRPC-1

#### 2.1.1. Cell Culture Preparation

Ascorbic acid can stimulate type I procollagen mRNA and collagen synthesis in the mouse calvaria-derived cell line MC3T3-E1, which was used to study the role of collagen synthesis in osteoblast differentiation [11]. Rat dental pulp MRPC-1 cells [12] were incubated in Dulbecco’s modified eagle medium (DMEM, ICN Biomedicals, Costa Mesa, CA, USA) containing 10% fetal bovine serum (FBS), 100 IU/mL penicillin, and 100 μg/mL streptomycin (P/S). Cells in the third or fourth passages were used. After six days, cells were harvested by digestion with 0.4 mL of 1X trypsin and 0.02% EDTA at 37°C for 10 min and transferred to fresh medium. Cell concentrationswere measured with a hemacytometer (Hemacytometer, Bright-line, Reichart, Buffalo, NY, USA) and adjusted to 200,000 cells/mL in DMEM with 10% FBS and P/S (100 IU/mL and 100 μg/mL).

#### 2.1.2. Preparation of PP and Agarose Gel Beads Containing PP

Native rat PP was kindly provided by Mary E. Marsh (University of Texas Dental Branch at Houston, TX, USA) and the preparation of native rat PP was described previously [6,7]. Multiple DSP-PP transcripts are present in rat [13]. Rat DSP-PP_523_ is the dominant transcript, which generated rat PP with 523 amino acids [14]. One percent and 1.2% agarose were separately prepared by dissolving either 30 or 36 mg dry agarose powder (Sigma, St. Louis, MO, USA) into 3mL deionized water, heated at 100 °C for 10 s, and then kept in a 60 °C water bath. Five groups of cells were used: (1) control, cell culture without agarose gel; (2) Group 1, agarose-no PP; (3) Group 2, agarose-0.2 μg PP; (4) Group 3, agarose-1 μg PP; and (5) Group 4, agarose 5 μg PP. In Groups 1–4, 4 μL of these solutions from each group were dropped into the centers of 12-well culture plates and allowed to solidify. In each group, 1.0 mL of cell suspension (200,000 cells/mL) was added. In the no agarose control group, the same amounts of cells were added. The cell culture plates were incubated at 37 °C under 5% CO_2_: 95%air and 100% relative humidity. The medium was changed every other day.

#### 2.1.3. Release of Native PP from Agarose-1 μg PP Beads

Agarose containing 1 μg native PP was cultured in DMEM medium with 10% FBS and P/S (100 IU/mL and 100 μg/mL)and the culture medium was collected on Day 1 (24 h), and Day 2 to Day 4 to test the PP release. Because PP was soluble in 5% trichloroacetic acid (TCA), the collected cultured medium was treated with 5% TCA, which precipitated the non-PP proteins in the medium [6]. The precipitated proteins were removed by centrifugation. We used this feature to isolate acidic proteins such as DSP and PP from culture medium [1] and identified these proteins by Stains-All staining, Western blot and Mass spectroscopic analyses [1]. The TCA-soluble portion was further neutralized with one-fifth original volume of 3 MTris Hydrochloride (Tris-HCl), pH 8.8 buffer, and precipitated with one-tenth volume of 1 M CaCl_2_. The precipitate was then dissolved in 0.1 M EDTA and loaded onto polyacrylamide gel for electrophoresis. The gels were stained with Stains-All to detect the presence of PP.

#### 2.1.4. Cell Migration Analyses and Immunohistochemistry Analyses on MRPC-1 Cells

To examine the cell migration under the influence of various agarose bead concentrations (0, 0.2, 1 and 5 μg) of PP, each group with three wells was observed on Day 2 under microscope. The cells located next to the agarose beads were counted in each group. The cells in the control group with no agarose were also counted randomly.

Six more wells in each group at Day 2were fixed with 4% form aldehyde. Three of these wells were used to detect collagen type I (Col I) expression with anti-collagen type I (Col I) antibodies at 200X dilution in 1% blocking agent with maleic acid in no agarose cell culture, and in Group 1 (agarose-no PP), Group 3 (agarose-1 μg PP) and Group 4 (agarose-5 μg PP) cell cultures. Next, diluted secondary biotinylated antibody was added and then shed and quenched of endogenous peroxidase activity. Finally, Vectastain Elite ABC reagent was added to the cultures and four drops of DAB stock solution (Vector laboratories) were added to each well for color observation. Following a similar protocol, another three wells were used to detect PP expression with anti-PP antibodies at 100X dilution in 1% blocking agent with maleic acid in four groups (i.e., agarose-no pp, agarose-0.2 μg PP, agarose-1 μg PP and agarose-5 μg PP).

Three wells from each group at Day 4 were fixed with 4% formaldehyde and used to detect PP expression with anti-PP antibody as described above.

### 2.2. Effect of Recombinant DSP_307_ Protein and DSP/PP_240_ Protein Mixture on Rat M2H4 Dental Pulp Cells

#### 2.2.1. Recombinant Protein Preparation

Rat DSP-PP_240_ cDNA in a baculovirus expression vector pVL1392 was used to generate recombinant DSP_430_/PP_240_ (i.e., DSP protein with 430 amino acid residues and PP protein with 240 amino acid residues) proteins in the insect sf9 cell culture medium [1]. Rat DSP_307_ cDNA was derived from a naturally occurring DSP-only transcript present in tooth germs [15]. Recombinant DSP_307_ protein (i.e., DSP protein with 307 amino acid residues sharing 286 amino acids with DSP_430_ protein) was also generated in a baculovirus system, and secreted into the insect culture medium (data not shown). These sf9 cell supernatants, from either anti-sense DSP-PP_240_ cDNA infected sf9 cell conditioned medium containing no recombinant proteins or sense-infected DSP_307_ cDNA or DSP-PP_240_ cDNAsf9 cells containing recombinant DSP_307_ or DSP_430_/PP_240_ protein mixture, were used directly for this study.

#### 2.2.2. Effect of Recombinant DSP/PP on Rat Dental Pulp M2H4 Cell Proliferation

When we were ready to test recombinant DSP/PP effects on dental pulp proliferation, MRPC-1 cells did not function well in Ritchie lab. Thus, we developed a M2H4 cell line [16], which was selected from single-cell cloning of the rat dental pulp cell line RPC-C2A [17] by screening confluent single-cell cultures for their ability to undergo mineralization. 

M2H4 cells (8 × 10^5^ cells/T-25 flask) were cultured in 5mL of M2H4 growth medium (i.e., Eagle’s minimum essential medium (EMEM) containing 100 IU/mL penicillin and 100 ug/mL streptomycin (P/S) and 10% fetal bovine serum (FBS)). Once the cells reached 90% confluency, 1 mL of conditioned medium (i.e., from either anti-sense DSP-PP_240_ cDNA-infected sf9 cells containing no recombinant protein or from sense DSP-PP_240_ cDNA-infected cells containing recombinant DSP_430_/PP_240_ protein mixture) and/or ascorbic acid (50 μg/mL) was added to the flasks separately. The flasks were changed every other day with the appropriate conditioned medium. Cells were treated with 1X trypsin-EDTA. The cell number per flask was determined by hemocytometer. Once cells were treated with 1X trypsin-EDTA at Day 2, Day 4 and Day 6, the flasks were discarded. Data are presented as the mean ±S.D. of triplicate samples.

#### 2.2.3. Detection of DSP-PP mRNA expression in rat dental pulp M2H4 cells cultured with DSP/PP_240_

M2H4 cells (8 × 10^5^ cells/T-25 flask) were cultured in 5mL of M2H4 growth medium. Once the cells reached 90% confluency, 1 mL each of: (1) condition medium (containing no recombinant protein); (2) condition medium with 50 μg/mL ascorbic acid; and (3) recombinant DSP/PP_240_ protein mixture plus 50μg/mL ascorbic acid, was added to the flask separately. The flasks were cultured for a total of eight days. The flasks were changed every other day with appropriate medium. Triplicate samples were for each group.

To test whether these treatment conditions would affect DSP-PP mRNA expression, the total RNAs from M2H4 cells in the different treated groups were reverse transcribed with oligo-d(T) primer/random primer and reverse transcriptase to generate cDNA pools for PCR analyses. DSP-PP upper primer (5’GAAGCATGTCCTTCTG3’) and DSP-PP lower primer (5’CTATCCTGCTGTGTCC3’) sequences were used to generate a 220bp DSP-PP DNA fragment. G3PDH upper primer (5’TGTTTGTGATGGGTGTGAACC3’) and lower primer (5’ACAGTCTTCTGAGTGGCAGT3’) sequences were used to generate a 175 bp PCR G3PDH DNA fragment was used as an internal experimental control. RT-PCR amplification was performed as follows: denaturation for 30s at 94 °C, annealing for 30s at 55 °C and extension for 1 min at 72 °C, for 30 cycles. The PCR products were analyzed by gel electrophoresis with Ethidium Bromide staining.

#### 2.2.4. M2H4 Cell Morphology Changes under Different Culture Medium 

M2H4 cells (8 × 10^5^ cells/T-25 flask) were cultured in 5mL of M2H4 growth medium as described in the previous section. Once the cells reached 90% confluency, 1 mL of conditioned medium was added to the following groups of T-25 flasks: (1) conditioned medium containing no recombinant protein;(2) conditioned medium containing no recombinant protein with ascorbic acid (50 μg/mL); (3) conditioned medium containing recombinant DSP_307_ protein; and (4) conditioned medium with recombinant DSP_430_/PP_240_ protein mixture. Medium from these cells was changed every other day for eight days. Triplicates were set up for each group. At Day 8, the cells from each group were examined by light microscopy.

### 2.3. Statistical Analysis

Results are presented as means ± standard deviation (S.D.). Two-sample *t*-test for mean difference with unequal variances was carried out using the program Statistical Analysis System (SAS Institute Inc., Cary, NC, USA) by personnel at the Center for Statistical Consultation and Research Center of the University of Michigan.

## 3. Results

### 3.1. PP Effects on Rat Dental Pulp MRPC-1 Cells

#### 3.1.1. PP Release from Agarose Beads

**Figure 1:**
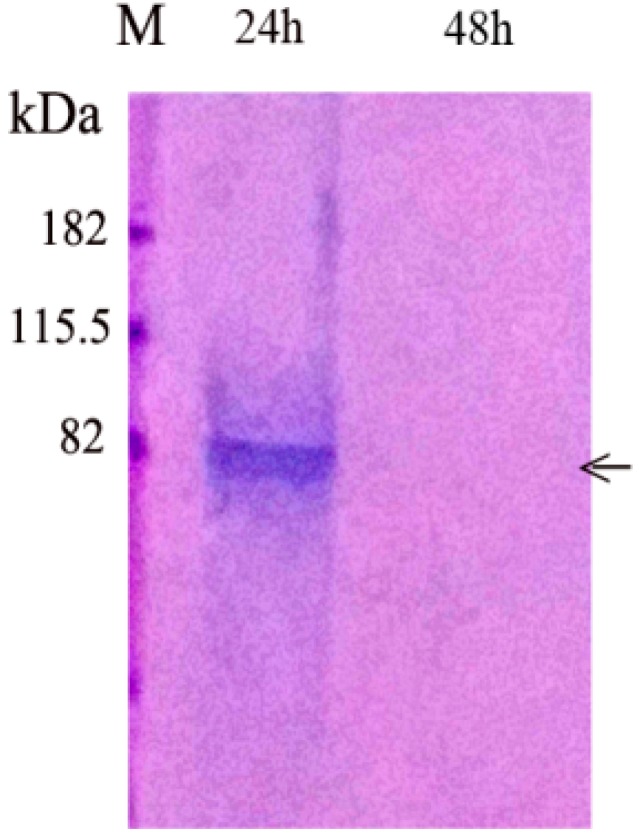
Release of native PP from agarose beads into culture medium. Agarose containing 1 μg native PP was cultured in DMEM medium with 10% FBS and P/S (100 IU/mL and 100 μg/mL). The culture medium was collected and PP release from agarose beads was measured. Culture medium collected at 24 h and 48 h were processed as described in Section 2.1.3. and analyzed using polyacrylamide gels then stained with Stains-All. M, molecular weight marker. Medium collected at 24 h contained PP as indicated by an arrow. Medium collected at 48 h did not contain PP.

To determine the rate of PP release from the agarose gel, agarose beads contained 1 μg of PP were cultured in DMEM with 10%FBS and P/S (100 IU/mL and 100 μg/mL). The culture medium was collected on Day 1 (24 h), and Day 2 to Day 4. We found that PP was completely released in 24 h into the culture medium. No further PP release was detected after 24 h (Figure 1). After the culture medium was changed on Day 2, no more PP was present in the cell culture medium.

#### 3.1.2. Rat Dental Pulp MRPC-1 Cell Migration Analysis

**Figure 2:**
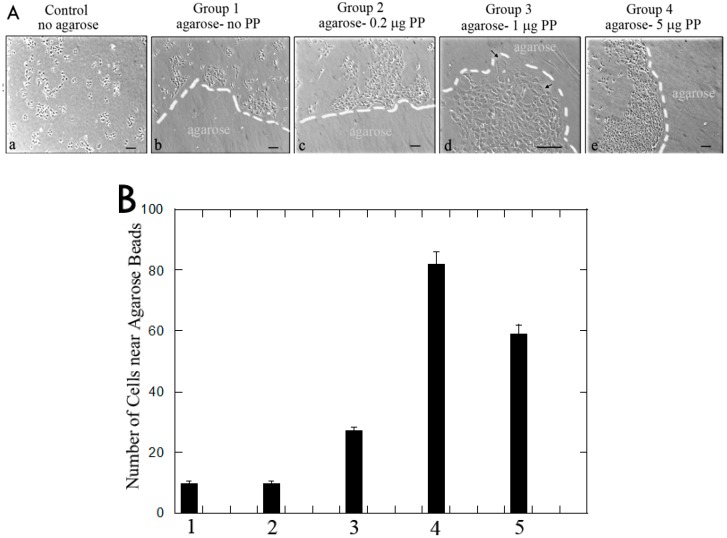
(**A**) Rat dental pulp MRPC-1 cell migration on Day 2:(**a**) scattered distribution of pulp cells in control group (i.e., no agarose in the well); (**b**) Group 1 (agarose-no PP), cells scattered around the agarose gel; (**c**) Group 2 (agarose-0.2 μg PP), cells slightly clustered around the agarose gel; (**d**) in Group 3 (agarose-1 μg PP), more cells were around the gel and wide spread cytoplasm was noted (indicated by arrows); and (**e**) in Group 4 (agarose-5 μg PP), more cells circled the gel. White dashed lines represent the boundary between cells and agarose bead. Scale bar = 100 μm for all frames. (**B**) Cell migration: Data from cell migration from each group are represented here as a bar graph. Error bars represent S.E. (*n* = 3).

Since PP was fully released into the culture medium within 24 h, we examined three wells from each group on Day 2. In general, all three wells for each group were examined under the microscope (Figure 2A). The control group without agarose beads showed sparse spindle-shaped pulp cells. Group 1 (agarose-no PP) displayed scattered cells around the agarose gel. Group 2 (agarose-0.2 μg PP) showed slightly clustered cells near the agarose border. Group 3 (agarose-1 μg PP) showed many cells surrounding the agarose beads. In Group 4 (agarose-5 μg PP), many cells encircled the agarose gel beads. The cell numbers located near the agarose beads in different groups were counted and presented as a bar graph (Figure 2B). The cell numbers from the control group (no agarose) were counted. The numbers of migrated cells present next to the PP-agarose gel were roughly proportional to PP concentrations (from no PP, 0.2 μg PP to 1μg PP) in the agarose gel (Figure 2B). However, the cell migration was decreased at agarose-5 μg PP group (Figure 2B).

#### 3.1.3. Col I and PP Expression in Rat Dental Pulp MRPC-1 Cells

**Figure 3:**
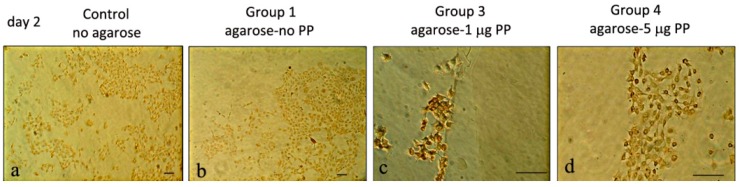
Col type I expression on Day 2 in rat dental pulp MRPC-1 cells:(**a**) cells in control group (no agarose) showed weak anti-Col I activity; (**b**) border of Group 1 (agarose-no PP) also showed weak anti-Col I activity. The cells were scattered around the gel; (**c**) cells on the border of Group 3 (agarose-1 μg PP) showed strong anti-Col I activity; and (**d**) cells around the border of Group 4 gel (agarose-5 μg PP) showed mild anti-Col l activity. Scale bar = 100 μm for all frames.

Using anti-Col I antibodies, immunohistochemistry showed weak Col I expression in control (no agarose) cultures and in Group 1 (agarose-no PP). Strong Col I expression in Group 3 (agarose-1 μg PP) and less Col I expression in Group 4 (agarose-5 μg PP). Overall, strong Col I expression appeared in Day 2 cells bordering Group 3 (agarose-1 μg PP) agarose beads (Figure 3).

**Figure 4:**
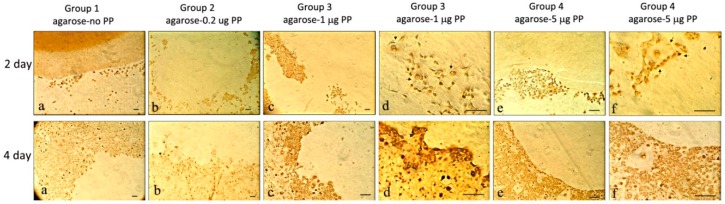
Anti-PP activities on Day 2 and Day 4 on rat dental pulp MRPC-1 cells. On Day 2: (**a**) cells were scattered around the agarose gel with less stain; (**b**) cells surround the gel with less stain; (**c**,**d**) more cells surround the gel and anti-PP activity was detected; and (**e**,**f**) cells in Group 4 (agarose-5 μg PP) surround the border of agarose gel and expressed anti-PP activity. On Day 4: (**a**) cells proliferated and encircled the agarose gel and no significant anti-PP activity was detected; (**b**) cells near the agarose border expressed weak anti-PP activity; (**c**) strong anti-PP activity was present in the cells around the gel; (**d**) cells around the agarose gel expressed strong anti-PP activity; and (**e**,**f**) cells encircled the border of agarose gel expressed anti-PP activity. Scale bar = 100 μm for all frames.

Using anti-PP antibodies, the PP expression was more intense (Figure 4) than that of Col I (Figure 3). For example, on Day 2, cells in Group 3 (agarose-1 μg PP) showed strong PP expression. In Group 4 (agarose-5 μg PP), the cells encircling the agarose gel showed relatively strong PP expression. On Day 4, cells in Groups 1 (agarose-no PP), 2 (agarose-0.2 μg PP), and 4 (agarose-5 μg PP) were weakly stained. Overall, PP expression appeared be strongest in Group 3 on Day 4. In addition, more PP staining was observed in the cell nuclei on Day 2, while more PP staining was localized in the cytoplasm on Day 4.

### 3.2. Recombinant DSP/PP_240_ Protein Effects on M2H4 Cells 

#### 3.2.1. Recombinant DSP/PP_240_ Protein Effect on M2H4 Cell Proliferation

**Figure 5:**
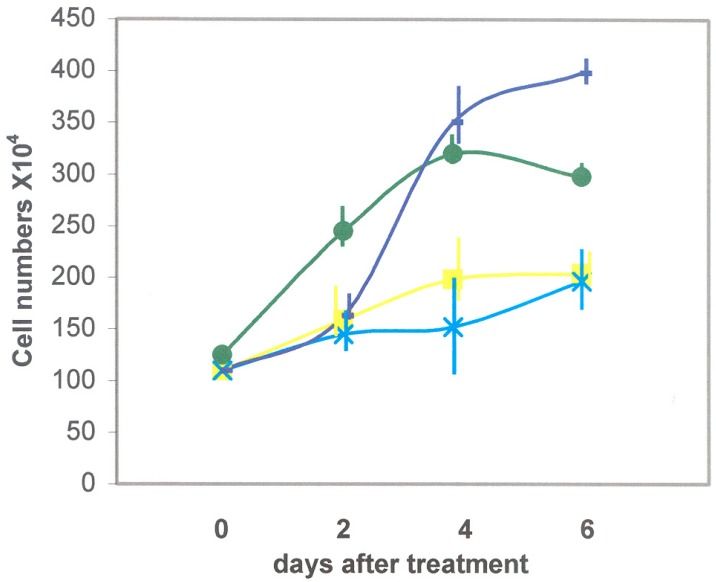
Effect of recombinant DSP/PP proteins on cell proliferation in rat dental pulp M2H4 cells. Dark blue line represents cells treated with sf9 conditioned medium containing anti-sense protein (no recombinant protein). Dark green represents cells treated with sf9 conditioned medium and ascorbic acid (50 μg/mL). Yellow line represents cells treated with DSP/PP recombinant proteins. Light blue line represents cells treated with DSP/PP recombinant proteins and ascorbic acid (50 μg/mL). Error bars represent S.E. (*n* = 3).

To test whether DSP and PP proteins could alter M2H4 dental pulp cell developmental programs, we first sought to determine whether recombinant DSP and PP proteins could alter M2H4 cell proliferation. Cells were incubated for six days in anti-sense conditioned media ± ascorbic acid, as well as sense conditioned media containing recombinant DSP/PP_240_ protein mixture± ascorbic acid. Figure 5 demonstrates that cell proliferation was most pronounced when M2H4 cells were incubated in the presence of anti-sense conditioned medium (i.e., containing no recombinant protein), while cell proliferation was slightly reduced in the presence of ascorbic acid. When M2H4 cells were cultured in the same medium with the presence of recombinant DSP/PP_240_ protein mixture, cell proliferation was significantly reduced. Similarly, in the presence of DSP-PP_240_ and ascorbic acid treatment, M2H4 proliferation was further reduced, as shown in Figure 5. 

#### 3.2.2. Recombinant DSP/PP_240_ Protein Mixture Plus Ascorbic Acid Up-Regulated DSP-PP mRNA Expression in M2H4 Cell Cultures 

**Figure 6:**
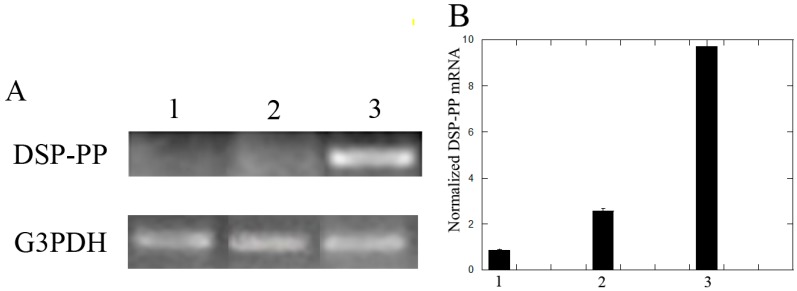
Effect of recombinant DSP/PP_240_ proteins on DSP-PP mRNA expression in M2H4 cells. Ninety-percent confluent M2H4 cells in T-25 flasks were treated for eight days with: (1) sf9 conditioned medium (CM); (2) conditioned medium with ascorbic acid (50 μg/mL) (CM + AA); and (3) recombinant DSP/PP_240_ proteins with ascorbic acid (50 μg/mL) (DSP/PP + AA).Cell medium plus treatment was changed every other day.(**A**) Polymerase chain reaction analyses of DSP-PP and G3PDH mRNA expression. (**B**) Bar graph of normalized DSP-PP mRNA expression under three different treatments. “1” represents CM, “2” represents CM + AA, and “3” represents DSP/PP + CM. Error bars represent S.E. (*n* = 3).

To determine the effect of recombinant DSP/PP_240_ proteins on DSP-PP mRNA expression in M2H4 cells, we compared its effect with those of sf9 condition medium in the presence or absence of ascorbic acid. We found that sf9 insect conditioned medium (without ascorbic acid) showed weak DSP-PP mRNA expression. In the presence of ascorbic acid, DSP-PP mRNA expression was slightly elevated. However, the addition of both recombinant DSP/PP_240_ and ascorbic acid to M2H4 cells significantly up-regulated DSP-PP mRNA expression (Figure 6).

#### 3.2.3. M2H4 Cell Morphology Changes That Occurred When Cultured in Various Medium 

**Figure 7:**
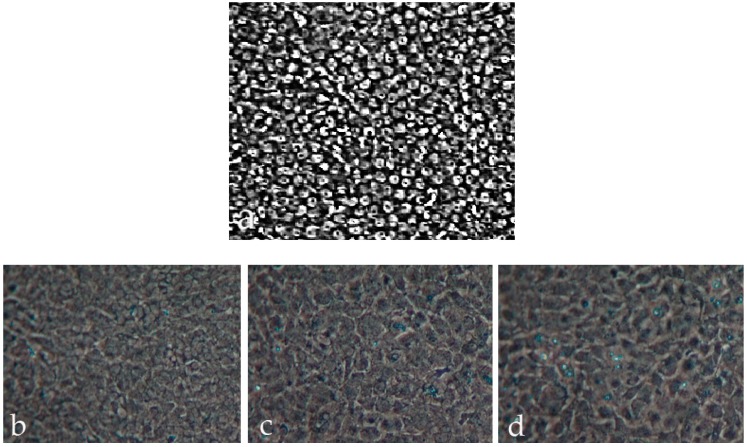
M2H4 cell morphology with different culture medium: (1) no recombinant protein; (2) ascorbic acid; (3) recombinant DSP_307_ protein; and (4) recombinant DSP_430_/PP_240_ protein mixture. (**a**) M2H4 cells cultured with conditioned medium (containing anti-sense DSP-PP cDNA; no recombinant protein) without ascorbic acid, showed highly packed rounded cells;(**b**) M2H4 cells with conditioned medium and ascorbic acid showed cells were spread out;(**c**) M2H4 cells with recombinant DSP_307_ protein showed more spread out cell structure; and (**d**) M2H4 cells with recombinant DSP/PP_240_ protein mixture showed more spread out cells. For details about each group treatment, please see Section 2.2.4.

M2H4 cells grown in growth medium containing sf9 anti-sense conditioned medium (no recombinant protein) for eight days showed highly packed rounded cells (Figure 7a). M2H4 cells grown for eight days in the same medium with 50 μg/mL ascorbic acid showed the appearance of spread out cells (Figure 7b). When M2H4 cells were incubated in recombinant DSP_307_ and DSP/PP_240_ conditioned medium, respectively, more spread out cells were observed in the cultures (Figure 7c,d ).

## 4. Discussion

Even though non-collagenous proteins (NCPs)account for less than 20% of dentin’s organic components, their participation in dentinal mineralization is well established. PP is the most abundant NCP in dentin [18]. Previous research has established that PP forms mineral aggregates which bind to collagen fibers to initiate dentin mineralization [8,19]. Our study examined the role of native PP’s influence on cell migration and on cell differentiation by monitoring the synthesis of pulp cells’ biomarkers such as Col I and PP.

Between the control group (no agarose) and Group 1 (agarose-no PP) at Day2, we found no difference between cell morphology and cell numbers.

In Day 2 cultures, MRPC-1 cells migrated slightly to the border of the agarose beads containing 0.2 μg PP. More cells migrated to the border of agarose beads containing 1 μg PP and cells completely encircled the agarose beads containing 5 μg PP (Figure 2). Thus, we conclude that PP promotes MRPC-1 cell migration. The numbers of cells present near the PP-agarose gel were roughly proportional to PP concentrations (0.2and 1 μg) in the agarose beads.

At higher PP concentration (5 μg), cell migration declined (Figure 2). On Day 2, the medium was replaced with fresh medium. We found that, after Day 2, no more PP was released from the agarose. Therefore, cells were not exposed to any PP protein. Thus, the cell migrations likely occurred during first 24 h in culture.

Col I is the major dentin matrix protein and provides the framework for the deposition of mineral crystals via interaction with non-collagenous proteins such as PP [8]. In this study, stronger Col type I expression appeared in the Day 2 cultures of the agarose-1 μg PP group than in the agarose-5 μg PP group. Thus, agarose-1 μg beads promoted better Col I expression.

Among various PP concentrations, the agarose-1 μg PP induced the most significant cell migration in MRPC-1 cells. The agarose-1 μg PP also exhibited a high level of Col type I expression on Day 2 and a high level of PP expression on Day 4. Taken together, agarose-1 μg beads promoted better cell migration, stronger Col I and PP expression.

The expression of Col I first followed by PP expression in MRPC-1 cells mimicked the Col I and PP expression patterns in vivo [20]. Likely the PP release from the agarose attracted cells to the beads (which occurred during the first 24 h). After Day 2, no more PP is available to the cells. Thus, the effects of PP on these migrated cells around the agarose beads are likely due to the first 24 h PP exposure. This PP exposure led to further differentiation of the MRPC-1 cells into odontoblast-like cells that expressed Col I and PP.

These results lend support that PP plays a role during epithelial–mesenchymal interactions. PP could be used to recruit dental pulp cells to the odontoblast layer during dentin formation and dentin repair. Thus, PP could potentially be used as a bio-mimetic material for tooth repair and tissue engineering.

Recombinant DSP/PP proteins reduced cell proliferation. Ascorbic acid has been reported to induce alveolar bone cell and dental pulp cell differentiation [11,16]. M2H4 cells treated with Sf9 anti-sense DSP-PP_240_ conditioned medium containing no recombinant protein displayed higher cell proliferation (i.e., 410 × 10^4^ cells) on Day 6 (Figure 5). M2H4 cells treated with ascorbic acid showed reduce cell proliferation on Day 4 and Day 6. The reduced cell proliferation likely suggested cell differentiation was now underway. Figure 6 showed that ascorbic acid treatment resulted in increased DSP-PP mRNA expression. Similar in vitro ascorbic acid requirements for cell differentiation have been observed for odontoblasts, osteoblasts and other mesenchyme-derived cells such as adipocytes, myocytes, and chondrocytes [21]. Cells treated with recombinant DSP/PP_240_ protein mixture showed reduced cell proliferation (i.e., 200 × 10^4^ cells) (Figure 5). Cells treated with recombinant DSP-PP_240_ protein mixture and ascorbic acid showed the lowest cell proliferation from Day 2 to Day 6 (Figure 5). The combination of recombinant DSP-PP_240_ protein mixture and ascorbic acid certainly promoted cell differentiation, as indicated by greatly increased DSP-PP mRNA expression (Figure 6).

Cells cultured in medium containing no recombinant protein and no ascorbic acid were rounded (Figure 7a), which agreed with the high cell proliferation that we measured (Figure 5). The presence of ascorbic acid seemed to cause cell spreading (Figure 7b). The cell spreading is likely due to cell differentiation caused by ascorbic acid treatment. The differentiated dental pulp cells likely actively synthesize extracellular matrix such as Col I and DSP/PP proteins. Furthermore, cell spreading appeared more prevalent when M2H4 cells were incubated in recombinant DSP_307_ and DSP/PP_240_ sense-conditioned media, respectively (Figure 7c,d). Both recombinant DSP_307_ and DSP/PP_240_ protein mixture might strongly promote dental cell differentiation and matrix synthesis.

## 5. Conclusions

In summary, our cell culture results suggest that native PP could promote dental pulp cell migration and induce cell differentiation causing Col I and DSP/PP protein expression. Recombinant DSP/PP proteins may also reduce cell proliferation. Taken together, these findings support our hypothesis that native PP and recombinant DSP/PP influence cell migration from dental pulp to the peripheral area, reduce proliferation and increase differentiation to enable pulp cells to develop into odontoblast-like cells and produce Col I and DSP/PP proteins.

These studies suggest the potential use of the native PP and the recombinant DSP/PP proteins as novel clinical capping agents for caries treatment.
